# Association of Anabolic Effect of Calcitriol with Osteoclast-Derived Wnt 10b Secretion

**DOI:** 10.3390/nu10091164

**Published:** 2018-08-25

**Authors:** Chien-Lin Lu, Jia-Fwu Shyu, Chia-Chao Wu, Chi-Feng Hung, Min-Tser Liao, Wen-Chih Liu, Cai-Mei Zheng, Yi-Chou Hou, Yuh-Feng Lin, Kuo-Cheng Lu

**Affiliations:** 1 Graduate Institute of Clinical Medicine, College of Medicine, Taipei Medical University, Taipei 11031, Taiwan; janlin0123@gmail.com (C.-L.L.); shyujeff@mail.ndmctsgh.edu.tw (J.-F.S.); wayneliu55@gmail.com (W.-C.L.); 11044@s.tmu.edu.tw (C.-M.Z.); linyf@shh.org.tw (Y.-F.L.); 2 Division of Nephrology, Department of Medicine, Fu Jen Catholic University Hospital, School of Medicine, Fu Jen Catholic University, New Taipei City 242, Taiwan; 3 Department of Biology and Anatomy, National Defense Medical Center, Taipei 114, Taiwan; 4 Division of Nephrology, Department of Medicine, Tri-Service General Hospital, National Defense Medical Center, Taipei 114, Taiwan; wucc@ndmctsgh.edu.tw; 5 School of Medicine, Fu-Jen Catholic University, New Taipei City 242, Taiwan; skin@mail.fju.edu.tw; 6 Graduate Institute of Biomedical and Pharmaceutical Science, Fu-Jen Catholic University, New Taipei City 262, Taiwan; 7 Department of Pediatrics, Taoyuan Armed Forces General Hospital, Taoyuan 325, Taiwan; liaoped804h@yahoo.com.tw; 8 Department of Pediatrics, Tri-Service General Hospital, National Defense Medical Center, Taipei 114, Taiwan; 9 Division of Nephrology, Department of Internal Medicine, Tungs’ Taichung MetroHarbor Hospital, Taichung City 433, Taiwan; 10 Department of Internal Medicine, School of Medicine, College of Medicine, Taipei Medical University, Taipei 11103, Taiwan; 11 Division of Nephrology, Department of Internal Medicine, Shuang Ho Hospital, New Taipei City 235, Taiwan; 12 Division of Nephrology, Department of Medicine, Cardinal-Tien Hospital, School of Medicine, Fu Jen Catholic University, New Taipei City 23155, Taiwan; athletics910@gmail.com

**Keywords:** bone turnover markers, calcitriol, hemodialysis, Wnt 10b

## Abstract

Canonical Wnt (Wingless/Integrated) signaling is crucial in bone development and the Wnt ligand can promote osteoblast differentiation from mesenchymal progenitor cells. Calcitriol, an active vitamin D3, is used clinically for treatment of secondary hyperparathyroidism (SHPT) in chronic kidney disease (CKD) patients. The bone effects of calcitriol in SHPT remains uncertain. We hypothesized that calcitriol improves bone mass by suppressing osteoclast activity, and simultaneously promoting Wnt ligand secretion. We designed a cross-sectional study in maintenance hemodialysis patients to explore the effects of calcitriol on different bone turnover markers and specifically emphasized the Wnt 10b levels. Then, we explored the source of Wnt 10b secretion by using osteoclasts and osteoblasts treated with calcitriol in cell culture studies. Finally, we explored the effects of calcitriol on bone microarchitectures in CKD mice, using the 5/6 nephrectomy CKD animal model with analysis using micro-computed tomography. Calcitriol promoted the growth of both trabecular and cortical bones in the CKD mice. Wnt 10b and Procollagen 1 *N*-terminal Propeptide (P1NP) significantly increased, but Tartrate-resistant acid phosphatase 5b (Trap 5b) significantly decreased in the calcitriol-treated maintenance hemodialysis patients. Calcitriol enhanced Wnt 10b secretion from osteoclasts in a dose-dependent manner. Treatment of SHPT with calcitriol improved the bone anabolism by inhibiting osteoclasts and promoting osteoblasts that might be achieved by increasing the Wnt 10b level.

## 1. Introduction

Canonical Wnt signaling is crucial in bone development, and the Wnt ligand can promote osteoblast and chondrocyte differentiation from mesenchymal progenitor cells [1]. Wnt proteins are a family of secreted proteins that regulate various stages of osteoblastogenesis and tightly control the process of bone remodeling [2]. Chondrocytes maturation requires canonical Wnt signaling, but the differentiation of mesenchymal progenitor cells toward the osteoblast lineage, rather than chondrogenic differentiation, occurs with adequate Wnt signaling promotion [3]. The canonical Wnt pathway suppresses osteoclastogenesis through increased osteoprotegrin (OPG) expression [4]. Peroxisome proliferator-activated receptor γ (PPARγ) is a key inducer of adipogenesis and inhibitor of osteoblastogenesis. Wnt signaling can inhibit PPARγ by repressing mRNA activity and inhibiting transactivation to suppress osteoclastogenesis [5,6].

Overexpression of Wnt 10b increases the number of osteoblasts per area of bone surface and the rate of mineral apposition results in increased bone volume fraction, trabecular number, and bone mineral density in the femoral bones of C57BL/6 mice [7]. Loss of Wnt 10b in mice causes age-dependent loss of trabecular bone due to a reduced number of mesenchymal progenitor cells (rather than altered osteoclast activity or number per trabecular bone area) [8]. Wnt 10b is considered to maintain mesenchymal or osteoblast progenitor cells required for postnatal bone homeostasis. Intermittently administered human parathyroid hormone (PTH; fragment 1–34) can increase bone mineral density and reduce the risk of fracture in osteoporosis [9,10], though the underlying mechanism remains unclear. Terauchi et al. [11] showed that PTH induces Wnt 10b production from bone marrow CD8+ cells and then activates canonical Wnt signaling in preosteoblasts. An association between two single nucleotide polymorphisms (SNPs) (rs1051886 and rs3741627) in the Wnt 10b gene and higher hip bone mineral density has also been confirmed [12]. 

Canonical Wnt signaling is activated when the Wnt ligand binds to a dual receptor complex comprising the Wnt co-receptor low density lipoprotein receptor-related protein (LRP) 5/6 and one of the seven transmembrane receptors of the Frizzled (FZD) family, disrupting the phosphorylation of β-catenin by adenomatous polyposis (APC) and glycogen synthase kinase 3β (GSK-3β). Dephosphorylated β-catenin translocates into the nucleus and binds T cell factor/lymphoid enhancer factor (TCF/LEF) transcription factors to initiate transcription. Sclerostin and Dickkopf-related protein 1 (DKK-1) antagonize Wnt signaling by binding to LRP 5, FRP 6, or both. These two Wnt inhibitors are produced by mature osteocytes embedded in the mineralized matrix. After sclerostin and DKK-1 are secreted in the osteocyte lacuna, they diffuse to the surface and inhibit osteoblastic bone formation.

Chronic kidney disease-mineral bone disease (CKD-MBD) is a spectrum of abnormalities of mineral metabolism and bone histomorphometric changes due to CKD. From early in disease progression, increased sclerostin expression is observed in juvenile cystic kidneys (jck) mice, which develop hyperparathyroidism and hyperphosphatemia. Repression of Wnt and β-catenin signaling increases the ratio of the receptor activator of nuclear factor kappa-B ligand (RANKL) to OPG, and subsequently enhances the activity of osteoclasts [13]. In patients with CKD, the PTH and bone expression of sclerostin are elevated in the earlier stages of disease, whereas phosphorylated β-catenin and FGF-23 are increased in late stages compared to healthy individuals [14]. These findings indicate that Wnt signaling plays an important role in the pathogenesis of renal osteodystrophy.

At present, in terms of bone clinical outcomes, such as bone fractures and bone pains, no benefit was observed from the administration of vitamin D compounds. However, small patient numbers and insufficient follow-up time could not ascertain these outcomes [15,16,17]. Among the severe SPTH group, osteitis fibrosa is a typical bone presentation of renal osteodystrophy. Calcitriol treatment can decrease bone turnover, bone resorption formation, and results in reduction of woven osteoid and fibrosis [4]. Mineralization and parameters of bone architecture improved [18,19]. Besides, long term intermittent calcitriol infusion (1–2.5 μg three times a week) increased the bone formation rate and osteoblastic osteoid, while ameliorating the degree of marrow fibrosis among severe osteitis fibrosa patients [20]. Therefore, the benefits of using calcitriol for SHPT is more prominent in higher PTH group, in whom it can both effectively lower the PTH levels and reverse the high bone turnover status to improve the bone mass. Based on these findings, we hypothesized that a therapeutic dose of calcitriol could increase bone mass, which might be achieved by directly suppressing osteoclasts and simultaneously promoting Wnt ligand secretion beyond the PTH lowering effects. We designed a cross-sectional study in maintenance hemodialysis patients to explore the effects of calcitriol on different bone turnover markers, specifically emphasizing the Wnt 10b levels. Then, we explored the source of Wnt 10b secretion by using osteoclasts and osteoblasts treated with calcitriol in in vitro cell culture studies. Finally, we explored the effects of calcitriol on bone microarchitectures in CKD mice, using 5/6 nephrectomy CKD animal model, with analysis via micro-computed tomography.

## 2. Materials and Methods

### 2.1. Clinical Study

#### 2.1.1. Patients

A total of 225 stable maintenance hemodialysis (HD) patients with HD for >3 months at Cardinal Tien Hospital (New Taipei City, Taiwan) and Shin Kong Memorial Hospital (Taipei City, Taiwan) were enrolled in our study (98 men, 127 women). The average age of study patients was 68.9 ± 12.1 years old (range 30 to 95 years old). We excluded subjects who switched to peritoneal dialysis, performed renal transplantation, with any sign of inflammation or infection, or malignancy history. This study was approved by the Human Ethical Committees of Shih Kong Memorial Hospital. The Institutional Review Board approval number was 20150814R. Written informed consent was obtained from all patients.

#### 2.1.2. Clinical and Laboratory Parameters

Clinical characteristics of the patients, including age and sex, were obtained from medical records. Blood samples were collected after overnight fasting and stored at −20 °C until analysis. Concentrations of plasma calcium, phosphate, albumin, hemoglobin, parathyroid hormone (PTH), and alkaline phosphatase (ALP) were measured with an automatic chemistry analyzer (Synchron LXi-725; Beckman Coulter Inc., Brea, CA, USA). The serum was also separated for testing bone turnover marker, including Wnt 10b, Wnt 16, β-catenin, sclerostin, Dickkopf-related protein 1(DKK-1), Tartrate-resistant acid phosphatase 5b (Trap 5b), free receptor activator of nuclear factor kappa-B ligand (RANKL), osteoprotegerin (OPG), Procollagen 1 *N*-terminal Propeptide (P1NP), Fibroblast growth factor 23 (FGF-23), and 25-hydroxyvitmain D (25-(OH)D), which were measured using commercially available ELISAs. (USCN: Wnt 10b, P1NP; Cusabio: Wnt 16; Abcam: β-catenin, sclerostin, DKK-1, FGF-23; Quiedl: Trap5b; Biomedica: Free RANKL, OPG; Immundiagnostik: 25-(OH)D).

### 2.2. Cell Culture Study

#### 2.2.1. Osteoclast Precursor Culture from Bone Marrow Monocyte 

The femoral and tibia bones were harvested from 8-week-old Sprague–Dawley rats and the attached connective tissue was removed. After epiphysis was removed, the remaining mid-shaft was minced and washed repeated in phosphate buffer solution (PBS) to remove marrow cells. Lymphocyte cell separation media (Lympholyte-H; Cedarlane Laboratories Ltd., Ontario, Canada) was used to separate the mononuclear cell layer at the interface after centrifuging at 400 g for 40 min. A total of 1 × 10^6^ monocytes was cultured in 6-cm dish in alpha modification of Minimum Essential Medium (α-MEM) (Gibco, Grand Island, NY, USA) medium containing 10% fetal bovine serum (FBS) (Gibco, Grand Island, NY, USA), 50 ng/mL macrophage colony-stimulating factor (M-CSF) (Rocky Hill, NJ, USA), and 50 ng/mL RANKL (Rocky Hill, NJ, USA) in the absence or presence of a 1 nm, 10 nM, and 100 nM dose of calcitriol overnight. The culture medium was changed every 3 days. 

#### 2.2.2. Preosteoblast Cell Line and Differentiation from Mature Osteoblasts

The 7F2 cell line was purchased from American Type Culture Collection (ATCC, CRK-12557, Manassas, VA, USA) and cultured in Dulbecco’s modified eagle medium (DMEM) (HyClone, Logan, UT, USA) supplemented with 10% fetal bovine serum(Gibco, Grand Island, NY, USA), 2 mM L-Glutamine (Nacalai Tesque, Kyoto, Japan), 1% sodium pyruvate (HyClone, Logan, UT, USA), and penicillin/ streptomycin (HyClone, Logan, UT, USA), at 37 °C in 6% CO_2_. To induce osteoblast differentiation, a total of 1 × 10^5^ 7F2 cells were plated in a 6-cm dish containing 100 μg/mL ascorbic acid (Sigma-Aldrich, St. Louis, MO, USA), and 10 mM β-glycerol phosphate (Sigma-Aldrich, St. Louis, MO, USA) in the absence or presence of a 1 nM, 10 nM, and 100nM dose of calcitriol (Sigma-Aldrich, St. Louis, MO, USA)overnight. The culture medium was changed every 3 days. 

#### 2.2.3. Osteoclast Wnt10b/Wnt16 Western Blotting Assay

Cell lysates were prepared by extracting proteins using a lysis buffer ((20 mM Tris-HCl, pH 7.5 150 mM NaCl, 1 mM ethylenediaminetetracetic acid (EDTA), 1 mM ethylene glycol tetraacetic acid (EGTA), 1% Triton, and 1 mM phenylmethylsulfony fluoride (PMSF)), supplemented with protease inhibitors. The proteins were separated using sodium dodecyl sulfate polyacrylamide gel electrophoresis (SDS-PAGE) and then transferred onto a polyvinylidene fluoride (PVDF) membrane (GE Healthcare Amersham^TM^, Piscataway, NJ, USA). The membrane was blocked with 5% nonfat dry milk in Tris-buffered saline and then incubated with the primary antibodies (Wnt 10b: ab 66721, abcam, Burlingame, CA, USA; Wnt 16: ab 64461, abcam, Burlingame, CA, USA) for 1 h at room temperature. The blots were then developed with a peroxidase-conjugated secondary antibody, and the proteins visualized using enhanced chemiluminescence procedures (Amersham, Arlington Heights, IL, USA) according to the manufacturer’s instructions.

#### 2.2.4. Tartrate-Resistant Acid Phosphatase (TRAP) Staining

Osteoclast were culture on 22 × 22-mm glass coverslips for 18 h and then treated with calcitriol (1 nM, 10 nM, or 100 nM) for 72 h. Then, cells were stained using the Sigma-Aldrich kit (Sigma-Aldrich, St. Louis, MO, USA), 387A, following the manufacture’s procedure. Osteoclast was identified with more than three nuclei. 

#### 2.2.5. Confocal Microscopic Analysis of Osteoclasts

Osteoclasts were cultured on 22 × 22 mm glass coverslips for 18 h. The osteoclasts were treated with 100 nM calcitriol for 16 h. After repeated cell washing by PBS, cells were fixed with 4% paraformaldehyde for 10 min and then permeabilized with 0.2% Triton X-100 for 5 min. Next, cells were incubated in 1% bovine serum albumin (BSA) (Sigma-Aldrich) in PBS for 1 h. The confocal image was analyzed by a microscope equipped with a differential interference contrast optical path (LSM 510, Zeiss, Göttingen, Germany). Cells with more than three nuclei were considered osteoclasts. An osteoclast was determined to contain an actin ring if more than half of the actin ring was labelled.

### 2.3. CKD Animal Study

#### 2.3.1. 5/6 Nephrectomy with Calcitriol Treatment

Male C57BL/6J mice aged 6–8 weeks were obtained from Bio-LASCO Taiwan Co., Ltd. (Taipei, Taiwan) and housed in a temperature-controlled room on a 12-h light/dark cycle. All animal procedures were approved by National Defense Medical Center Institutional Review Committee (Taipei, Taiwan). Mice had ad libitum access to food and water. Mice underwent 5/6 nephrectomy as described below. The nephrectomy was performed after intraperitoneal injection of sodium pentobarbital (50 mg/kg body weight). A left paravertebral cut was made in the dorsal region of the mouse then further dissection of skin, muscle, and adipose tissue was performed until left kidney was exposed. Lower branch of left renal artery was ligated with 4-0 silk to produce about 1/3 the area of kidney ischemia. Upper portion of left kidney was removed by cautery. Right side kidney was then nephrectomized by totally ligation of renal vessel and renal hilum with 4-0 silk to produce 5/6 nephrectomy. Finally, the retroperitoneum and muscle were closed with 4-0 nylon suture. Drug treatment was started one month after 5/6 nephrectomy.

Animals were divided into 3 groups: Group I was a control group, only 5/6 nephrectomy was performed and they did not receive calcitriol treatment (*n* = 3); Group 2 was an experimental group (5/6 nephrectomy) fed on calcitriol diluted with coconut oil at a dose of 25 IU/kg/day by oral gavage (*n* = 3); and Group 3 was another experimental group (5/6 nephrectomy) fed on calcitriol diluted with coconut oil at a dose of 150 IU/Kg/day by oral gavage (*n* = 3 ). All above drug treatments were given daily for a one-month duration and then animals were sacrificed. 

#### 2.3.2. Micro-Computed Tomography

The femoral bone microarchitecture was investigated using micro-computed tomography (CT) (SkyScan 1174; SkyScan, Aartselaar, Belgium). The area to be scanned was 4 mm proximal to the epiphyseal growth plate, with a pixel size of 4.59 μm. The X-ray source was operated at a 50 kV acceleration voltage with a Cu (40 μm) and Al (0.5mm) filter. All samples were scanned at a complete 360° rotation at a step size of 0.7° and under an exposure time of 301 milliseconds (ms). The trabecular bone was reconstructed and analyzed by using Skyscan software (Bruker, Kontich, Belgium) by drawing ellipsoid contours. 

### 2.4. Statistical Analysis

Continuous variables are expressed as means and standard deviation (SD). Normal distribution was evaluated by the Kolmogorov-Smirnov test and Shapiro-Wilk test. For continuous variables, the Student’s *t*-test was used for independent samples with normally-distributed values and the Mann-Whitney U-test was performed for values without normal distribution. For categorical variables, chi-square test and Fisher’s exact test were used. A two-tailed *p* value <0.05 was considered statistically significant. All analyses were performed with IBM SPSS Statistics version 20.0 (SPSS Inc., Chicago, IL, USA) for Windows.

## 3. Results

### 3.1. Clinical Study

The clinical characteristics of the HD patients are summarized in Table 1 and Table 2. The levels of serum PTH, alkaline phosphatase (ALP), and FGF-23 were higher, and albumin lower, in patients treated with calcitriol (Table 1). Regarding markers of bone turnover, the concentrations of Wnt 10b and P1NP were significantly increased in the calcitriol treatment group, but DKK-1 and tartrate-resistant acid phosphatase 5b (Trap 5b) were significantly decreased (Table 2). Sclerostin also decreased in the calcitriol treatment group (*p* = 0.06). Wnt 16, β-catenin, free RANKL, and osteoprotegerin (OPG) did not exhibit differences between HD patients with or without calcitriol treatment in the last three months. 

The correlation analysis for serum Wnt 10b levels and markers of bone turnover or clinical parameters in HD patients treated with calcitriol are provided in Table 3. A positive association was observed between Wnt 10b and Wnt 16, P1NP, PTH, and FGF23. Wnt 10b was negatively associated with sclerostin and Trap 5b. The serum 25D levels were not correlated with a change in Wnt 10b in patients treated with calcitriol.

**Figure 1:**
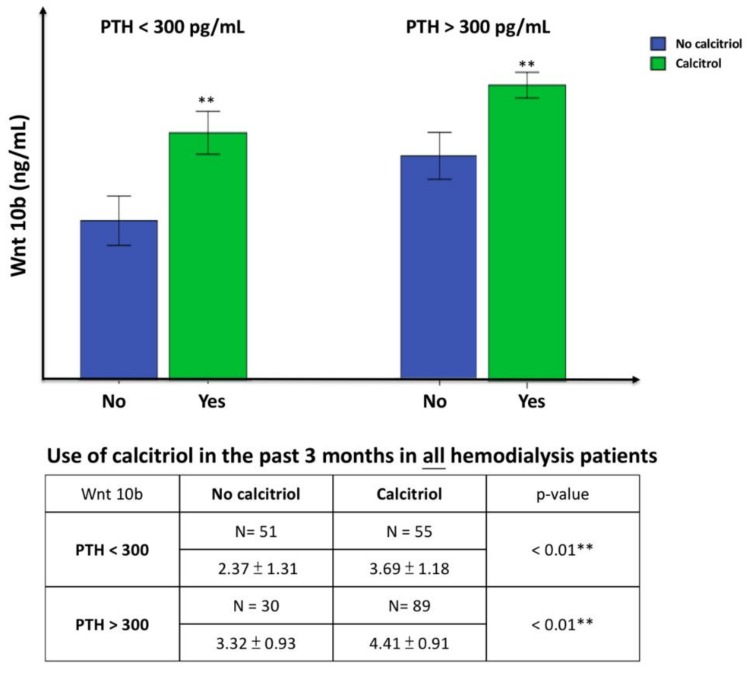
Comparison of serum Wnt 10b levels to the use of calcitriol in hemodialysis patients with PTH < 300 pg/mL group (left) and PTH > 300 pg/mL group (right). Values are means ± SD. (Blue: No calcitriol; green: Calcitriol use). In patients with PTH > 300 pg/mL, Wnt 10b was significantly greater in patients treated with calcitriol than those not treated with calcitriol. Because the PTH value was measured at the end of three months’ calcitriol treatment, there were 55 patients with PTH < 300 pg/mL given calcitriol. Wnt 10b was also significantly greater in patients treated with calcitriol than those not treated with calcitriol. ** *p* < 0.01.

In clinical practice, calcitriol is provided to treat SHPT in HD patients when PTH > 300 pg/mL. To clarify the independent impact of calcitriol on the expression of Wnt 10b, all HD patients were divided into two groups according to PTH level (PTH was measured at the end of three months’ calcitriol treatment), as shown in Figure 1. In patients with PTH > 300 pg/mL, Wnt 10b was significantly greater in patients treated with calcitriol than those not treated with calcitriol. Similar findings were obtained in patients with PTH < 300 pg/mL. 

**Figure 2:**
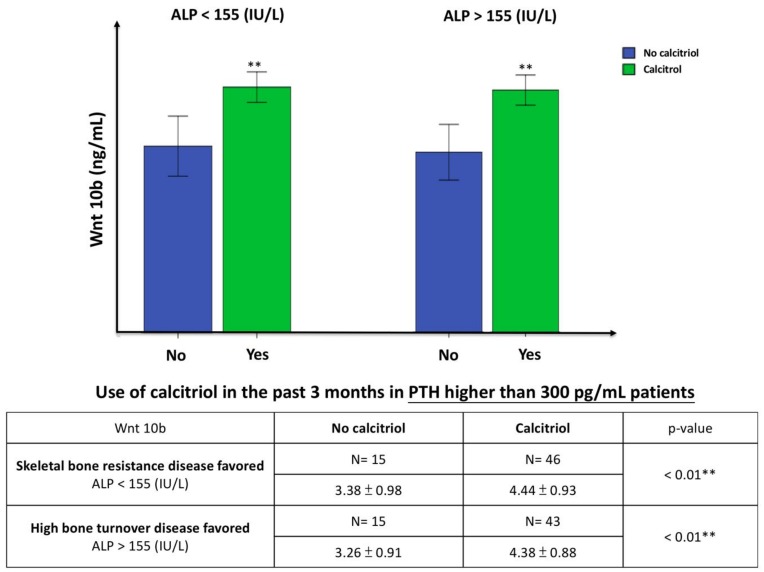
The comparison of serum Wnt 10b level to the use of calcitriol in patients with skeletal bone resistance disease favored (Left, PTH > 300 pg/mL and ALP < 155 IU/L) and high bone turnover disease favored (Right, PTH > 300 pg/mL and ALP > 155 IU/L). Values are means ± SD. (Blue: No calcitriol; green: Calcitriol). Wnt 10b was significantly increased in both skeletal bone resistance disease group and high bone turnover disease group with calcitriol treatment. ** *p* < 0.01.

**Figure 3:**
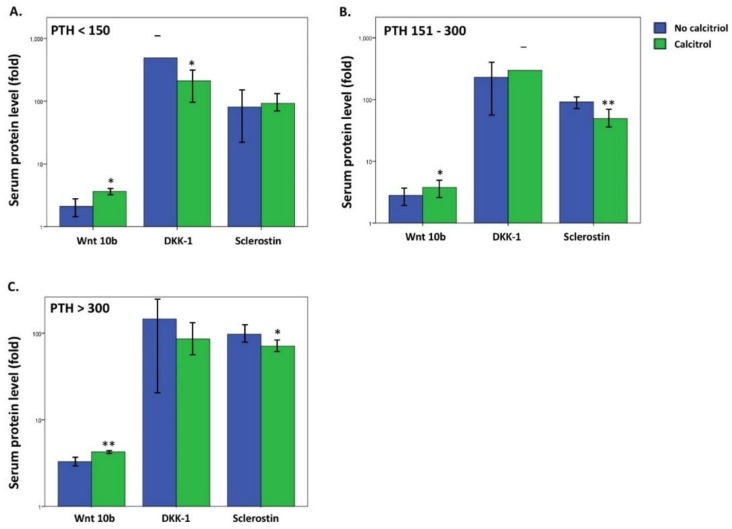
Comparison of serum protein level of Wnt inhibitors (DKK-1 and sclerostin) to the use of calcitriol in different PTH group. ((**A**). PTH < 150 pg/mL, (**B**). PTH 151–300 pg/mL, (**C**). PTH > 300 pg/mL.) Values are means ± SD. (Blue: No calcitriol; green: Calcitriol). DKK-1 was significantly lower in the PTH < 150 pg/mL subgroup ((**A**). *p* < 0.05) and sclerostin was significantly lower in both the PTH 151–300 pg/mL subgroup ((**B**). *p* < 0.01) and PTH > 300 pg/mL subgroup ((**C**). *p* < 0.05). * *p* < 0.05, ** *p* < 0.01.

Bone alkaline phosphatase (bALP) increased in HD patient with high bone turnover disease [21]. Combined high PTH and ALP may represent an alternative marker of high bone turnover disease in HD patients, and a lower level of ALP in HD patients may indicate skeletal resistance to PTH [22]. Figure 2 presents the Wnt 10b expression in HD patients with high bone turnover disease (PTH > 300 pg/mL and ALP > median value 155 IU/L) or skeletal resistance to PTH (PTH > 300 pg/mL and ALP < median value 155 IU/L). Wnt 10b significantly increased in the high bone turnover disease group with calcitriol treatment. Surprisingly, Wnt 10b levels were significantly higher in the PTH resistance group treated with calcitriol compared to those not treated with calcitriol (*p* < 0.01). DKK–1 and sclerostin are both upstream inhibitors of the Wnt pathway and the levels were significantly lower after calcitriol treatment in different PTH subgroups compared to the untreated subgroups: DKK–1 was significantly lower in the PTH < 150 mg/mL subgroup (*p* < 0.05) and sclerostin was significantly lower in both the PTH > 150 pg/mL (*p* < 0.01) and PTH > 300 pg/mL subgroups (*p* < 0.05) (Figure 3).

**Figure 4:**
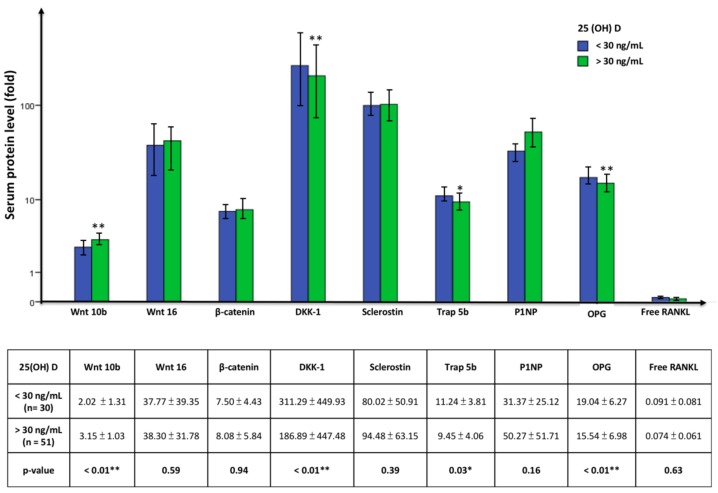
The comparison of serum bone turnover marker in patient without calcitriol use in 25(OH)D level < 30 ng/mL vs. > 30 ng/mL group. Values are means ± SD. (Blue: 25(OH)D < 30 ng/mL; green: 25(OH)D > 30 ng/mL). Patients with 25(OH)D > 30 ng/mL had significantly higher Wnt 10b levels but lower DKK-1, Trap 5b, and OPG levels compared to the group with 25(OH)D < 30 ng/mL. * *p* < 0.05, ** *p* < 0.01.

**Figure 5:**
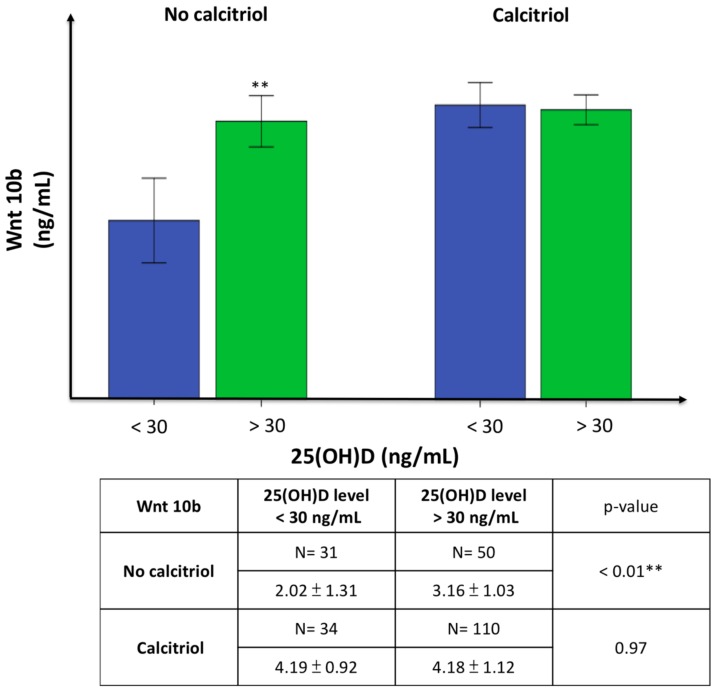
The comparison of serum Wnt 10b level to the serum 25(OH)D level in patients without (left) or with (right) calcitriol treatment. Values are means ± SD. (Blue: 25(OH)D < 30 ng/mL; green: 25(OH)D > 30 ng/mL). Wnt 10b was increased significantly in patients with 25(OH)D > 30 ng/mL if they did not receive calcitriol treatment in the past three months, but this significance was lost in the calcitriol treatment group. ** *p* < 0.01.

Vitamin D inadequacy is defined as serum 25(OH)D < 30 ng/mL and adequate levels as ≥30 ng/mL. We evaluated the effect of 25 (OH)D level on changes in bone turnover markers in patients not treated with calcitriol to avoid the effect of calcitriol on these markers. In the untreated group, patients with adequate 25(OH)D had significantly elevated Wnt 10b levels but lower DKK-1, Trap 5b, and OPG levels compared to the group with vitamin D inadequacy. P1NP also increased in the group with adequate vitamin D, though the difference was not significant (Figure 4). Furthermore, Wnt 10b increased significantly in patients with 25(OH)D > 30 ng/mL if they did not receive calcitriol treatment in the past three months, but this significance was lost in the calcitriol treatment group (Figure 5).

### 3.2. Cell Culture Study

**Figure 6:**
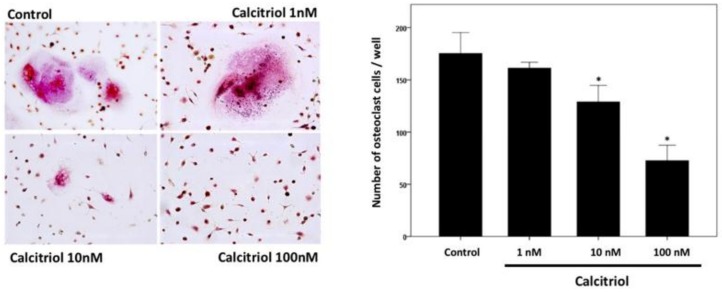
Tartrate-resistant acid phosphatase (Trap) stain of the osteoclast. Calcitriol inhibits the fusion ability and the number of TRAP-positive differentiated osteoclast cell in a dose-dependent manner. Multinuclear TRAP-positive cells containing more than three nuclei were scored as osteoclasts. Data are mean number ± SD of osteoclasts. (* *p* < 0.05 versus control group, *n* = 3).

**Figure 7:**
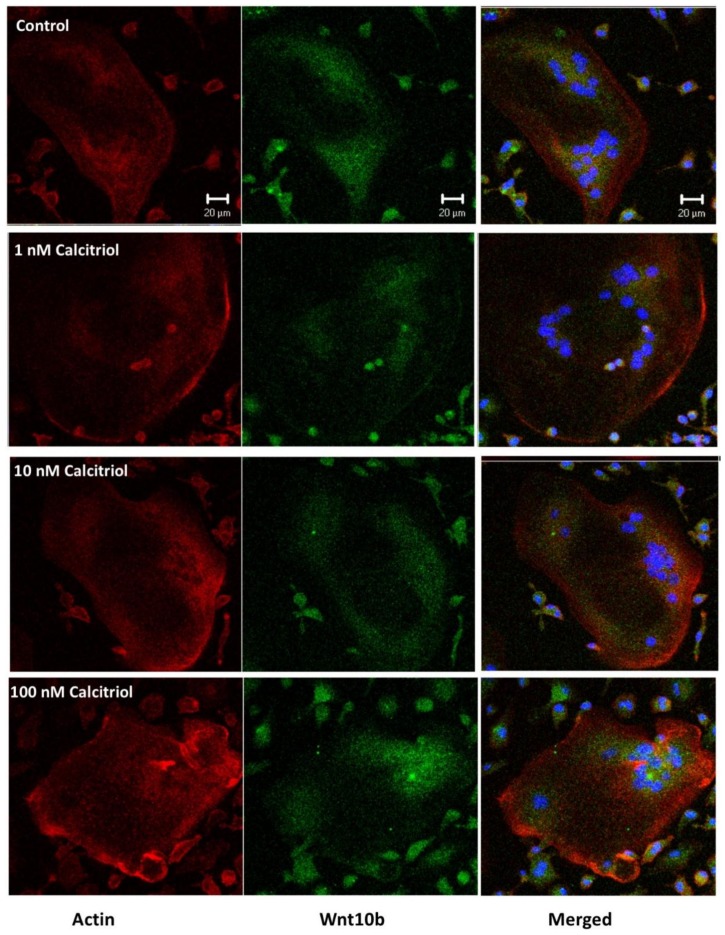
Confocal analysis of immunofluorescent labeling of Wnt 10b (green) was greater in osteoclasts treated with 1, 10, or 100 nM calcitriol compared to control. Actin was labeled with Cy3 (red). Bar = 20 μm.

**Figure 8:**
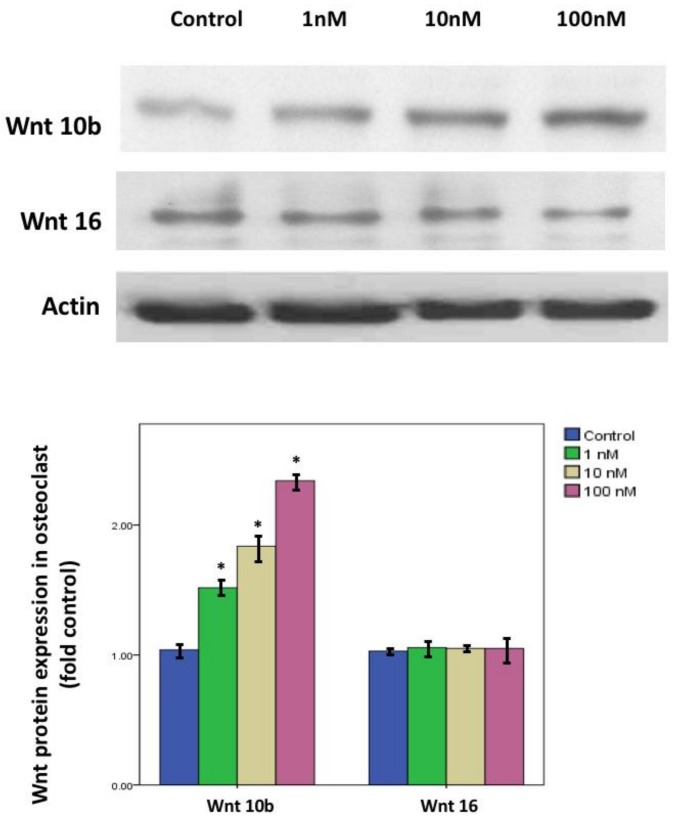
Wnt10b and Wnt 16 protein expression in calcitriol-treated osteoclasts. With calcitriol treatment of 10 to 100 nM overnight, Western blot analysis showed a significant increase in Wnt 10b expression. Wnt 16 expression in osteoclasts after different doses of calcitriol was not significantly different at the protein level. Actin protein served as a loading control. Data are means ± SD. (* *p* < 0.05 versus control group, *n* = 3).

In primary cell cultures of osteoclast stimulated with 50 ng/mL M-CSF and RANKL, calcitriol inhibited the fusion ability and the number of TRAP stain-positive differentiated osteoclasts in a dose-dependent manner (Figure 6). Confocal analysis of immunofluorescent labeling of Wnt 10b was greater in osteoclasts treated with 1, 10, or 100 nM calcitriol compared to control (Figure 7). With calcitriol treatment of 10 nM to 100 nM, Western blot analysis showed a significant increase in the Wnt 10b expression (Figure 8, upper). Wnt 16 expression in osteoclasts after different doses of calcitriol was not significantly different at the protein level (Figure 8, middle). 

**Figure 9:**
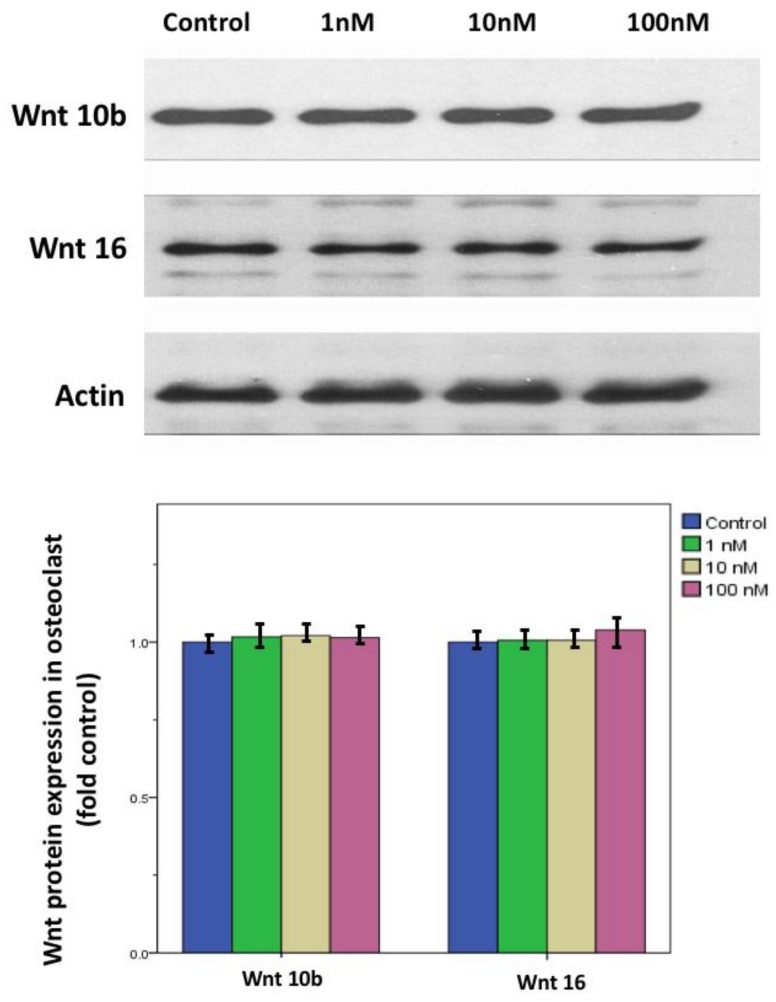
Wnt10b and Wnt 16 protein expression in calcitriol-treated osteoblasts. After preosteoblast 7F2 cells were stimulated with 100 μg/mL ascorbic acid and 10 mM β-glycerol phosphate, Western blot analysis showed both Wnt 10b and Wnt 16 expression in osteoblast at different doses of calcitriol was not significantly different at the protein level. Actin protein served as a loading control. Data are means ± SD (*n* = 3).

After preosteoblast 7F2 cell stimulated with 100 μg/mL ascorbic acid and 10 mM β-glycerol phosphate, Western blot analysis showed both Wnt 10b and Wnt 16 expression in osteoblast at different doses of calcitriol was not significantly different at the protein level (Figure 9).

### 3.3. CKD Animal Study

**Figure 10:**
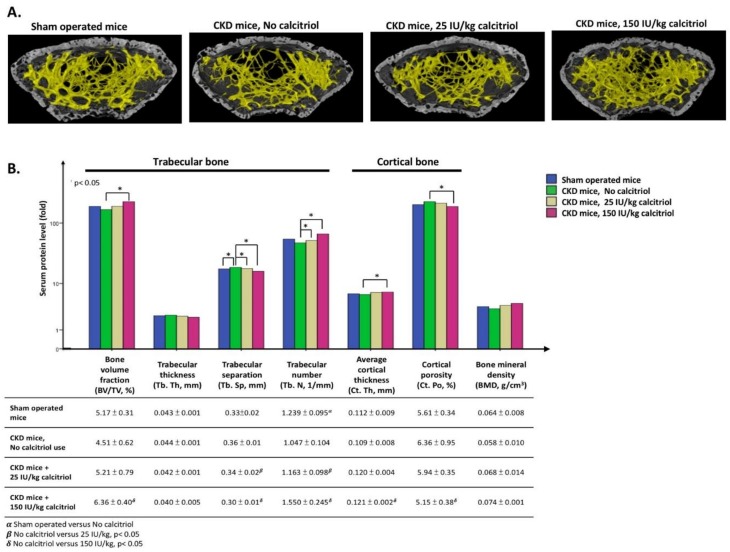
Three-dimensional (3D) image obtained via Bruker micro-tomography. (**A**) Trabecular bone mass (yellow part) of femur bone was greater after calcitriol treatment. (**B**) The growth of trabecular and cortical bone all increased significantly, especially calcitriol dose of 150 IU/kg for one month. (Blue: Sham operated control; green: CKD mice, No calcitriol; yellow: CKD mice, 25 IU/kg calcitriol use; red: CKD mice, 150 IU/kg calcitriol use). All experiments in this figure use mice in the C57/Bl6 background. (* *p* < 0.05, *n* = 3).

On three-dimensional (3D) images obtained by Burker micro-tomography, the trabecular bone mass (yellow part) of the femur bone was greater after calcitriol treatment (Figure 10, upper). In CKD mice treated with 25 IU/kg calcitriol, the trabecular separation significantly increased and trabecular number diminished compared to the control. The bone volume fraction reached significance with a calcitriol dose of 150 IU/kg for one month, but trabecular thickness was not significantly different in any group. Regarding cortical bone parameters, such as average cortical thickness and cortical porosity, a 150 IU/dose calcitriol had a significant effect compared to control, but this effect was not seen in the 25 IU/kg group. Overall, bone mineral density was unaltered in the 25 IU/kg and 150 IU/kg groups with such a short-term follow up (Figure 10, lower). 

## 4. Discussion

In physiological condition, 1,25-OH Vitamin D3 (calcitriol; reference 20–45 pg/mL) can stimulate osteoblast activity and following osteoclast differentiation (osteoclastogenesis) by RANKL expression. However, in most clinical scenarios, calcitriol used in treating secondary hyperparathyroidism (SHPT) requires relatively higher doses (μg/mL). This study focused on the change in serum Wnt 10b after receiving a therapeutic dose of calcitriol for SHPT. We found that calcitriol treatment significantly increased the level of Wnt 10b but not Wnt 16 in HD patients. Higher PTH and ALP reflect more severe SHPT that requires calcitriol treatment. FGF-23 also increased after calcitriol treatment because calcitriol can induce FGF-23 expression and secretion from bone [23] as well as enhance the gut absorption of phosphorus, further stimulating FGF-23 secretion [24]. 

As kidney function deteriorates, HD patients experience both protein-calorie malnutrition and inflammation attack, decreasing albumin synthesis. Pro-inflammatory cytokines, such as interleukin-1 (IL-1), stimulate Kupffer cells in the liver to produce IL-6 and enhance acute-phase reactants, such as C-reactive protein (CRP) and serum amyloid A (SAA), to subsequently suppress albumin synthesis [25]. In addition, hyperparathyroidism aggravates the inflammation status in HD, and PTH level is positively correlated with inflammation severity [26]. Thus, albumin was much lower in HD patients with higher PTH levels in this study. 

Regarding bone turnover activity, Trap 5b levels were significantly lower and P1NP levels significantly higher after calcitriol treatment. Thus, the response to calcitriol in bone metabolism was to inhibit osteoclasts and promote osteoblasts in our study. By interrupting the coupling effect of bone remodeling, calcitriol has more therapeutic windows for treating renal osteodystrophy. Increased osteoblast markers, osteocalcin, and decreased resorption marker Trap 5b have also been reported after calcitriol treatment [27]. This phenomenon can also be seen in the Pearson correlation analysis in the receiving group, which emphasizes the discrepancy effect of calcitriol. The Wnt inhibitors, sclerostin and DKK-1, diminished in the calcitriol treatment group and promoted the Wnt/β-catenin pathway in bone formation. In short, calcitriol enhanced bone formation by both increasing Wnt 10b and reducing the Wnt inhibitor-related β-catenin signaling pathway. Notably, lower sclerostin or DKK-1 levels benefited bone quality but not vessel calcification, as sclerostin or DKK-1 can be viewed as local counter-regulatory mediators suppressing vessel calcification [28,29]. Lower levels of sclerostin due to calcitriol treatment may increase the risk of vessel calcification. Therefore, the vessel calcification burden in HD patients must kept in mind during calcitriol treatment. 

Intermittent PTH can stimulate bone marrow CD8+ cells to produce Wnt 10b and activate the canonical Wnt pathway in pre-osteoblasts [11]. We also found that the Wnt 10b concentration was positively correlated with PTH regardless of calcitriol use in the past three months. As shown in Figure 2, calcitriol can directly stimulate Wnt 10b secretion without PTH.

A consensus was reached in discriminating different forms of renal osteodystrophy. Both PTH and ALP need to be used to evaluate underlying bone turnover [21]. Hyperparathyroid bone diseases are characterized by high PTH and high ALP levels. Low PTH and lower quartile laboratory reference range ALP levels have high positive predictive value for adynamic bone disease [30], and high PTH and low ALP levels indicate the presence of skeletal resistance. Accumulation of the 7–84 PTH fragment, increased OPG/RANKL ratio, and decreased PTH receptor in osteoblasts are all possible causes of skeletal resistance to PTH. Uremic toxins, such as indoxyl sulfate/p-cresol sulfate and phosphorus in CKD, are also aggravating factors resulting in PTH resistance. Skeletal resistance is related to high oxidant stress status [31] and the onset of SHPT [32]. Moreover, the effect of calcitriol-stimulated bone formation was further enhanced by the suppression of DKK-1 if PTH was less than 150 pg/mL and sclerostin if PTH was higher than 150 pg/mL. 

The activity of Wnt 10b was enhanced if 25(OH)D was adequate and the bone remodeling shifted to more bone formation with significantly lower Trap 5b levels and high P1NP levels. In addition, DKK-1 reduced accordingly. These findings indicate that maintaining adequate vitamin D levels in HD patients benefits bone metabolism and creating more healthy bone. Osteoblasts express the vitamin D binding protein receptors cubulin and megalin to uptake 25(OH)D and CYP27B1 mRNA that convert 25(OH)D to 1,25 (OH)_2_D intrinsically in response to 25(OH)D [33]. After 25(OH)D is given, bone formation marker genes, such as osteocalcin, osteopontin, and RANKL, are up-regulated [34]. Surprisingly, Wnt 10b was significantly prominent in the adequate 25(OH)D subgroup in the absence of using calcitriol. Thus, an adequate 25(OH)D level has a positive effect on Wnt 10b secretion, and this effect is covered if using calcitriol concurrently. 

Notably, the benefit of cholecalciferol supplementation on clinical end-point remains questionable. Some studies reported the effect of cholecalciferol supplement on vitamin D deficiency, PTH-lowering, and bone fracture or bone mineral density (BMD) in hemodialysis patients. Receiving cholecalciferol 25,000 IU every two weeks for one year can dramatically improve serum 25-vitmian D concentration and decrease PTH levels in HD patients [35]. Oral cholecalciferol 2000 IU three times per week for one year was effective in treating vitamin D deficiency in hemodialysis patients but was insufficient to lower PTH levels or improve BMD [36]. The effect of cholecalciferol appears to vary with the timing, dosage, and frequency of treatment. Therefore, larger randomized controlled trials with clinical meaningful endpoints such as fracture, BMD, hospitalization, or mortality, still must be evaluated in the HD population.

We hypothesize that Wnt 10b is a clastokine from osteoclasts, playing a role in communication between osteoclasts and osteoblasts, and then exerting its function to promote osteoblastogenesis via binding to FZD and LRP 5/6 co-receptor. Our study is the first to confirm that calcitriol inhibits the process of osteoclast maturation and fusion by using TRAP stain to identify the osteoclasts. This finding is compatible with lower Trap 5b levels in HD patients receiving calcitriol. Previous studies have shown that calcitriol inhibits osteoclastogenesis at the early stage of differentiation in a concentration-dependent manner [37]. The bone-resorbing activity of osteoclasts is also diminished at a 10 nM dose of calcitriol. The c-Fos protein is a target of calcitriol action in inhibiting osteoclastogenesis [38]. We also found that calcitriol enhances Wnt 10b secretion during osteoclastogenesis in a dose-dependent manner. Unlike Wnt 10b, Wnt 16 seems to be constitutively expressed in osteoclasts and is not associated with calcitriol treatment. In addition, Wnt 10b can also secreted from osteoblasts, which seem to be constitutively expressed in osteoblasts with an autocrine effect. One month of calcitriol in CKD mice showed that the fraction of a given volume occupied by the mineralized volume (BV/TV) significantly increased. The BMD also increased in the higher-dose calcitriol group. Calcitriol can promote the growth of both trabecular and cortical bone, especially in higher-dose calcitriol group. 

Taken together, the bone anabolic effect of a therapeutic dose calcitriol promotes osteoblast function and inhibits osteoclast viability in HD patients with SHPT. Increased Wnt 10b levels can explain this discrepancy in the effects of calcitriol on both osteoclasts and osteoblasts. Both osteoclasts and osteoblasts are the source of Wnt 10b, and therapeutic doses of calcitriol can enhance Wnt 10b secretion from osteoclasts in a dose-dependent manner.

Our study has several limitations: Firstly, the study size was small, and the total doses of calcitriol used in the three months were not calculated. Secondly, bone ALP is more suitable for assessing bone turnover status and provides more powerful predictive evidence to discuss the relationship between calcitriol and Wnt 10b. Thirdly, this is a cross-sectional study and we failed to explore the clinical outcome under calcitriol treatment. In the future, we would design a longitudinal prospective study to investigate the effect of calcitriol on various clinical end-points, such as bone fracture or bone mineral density. However, the results were promising and clinically important, and a multicenter interventional study with a larger number of subjects and multiple dialysis centers is needed.

## 5. Conclusions

The bone anabolic effect of a therapeutic dose calcitriol is relative to promoting osteoblast function and inhibiting osteoclast viability in HD patients with SHPT. Both osteoclast and osteoblast are the source of Wnt 10b, and a therapeutic dose calcitriol can enhance Wnt 10b secretion from osteoclasts in a dose-dependent manner. These findings suggest treatment of SHPT with calcitriol can improve bone anabolism through inhibiting osteoclasts and promoting osteoblasts, achieved by increasing Wnt 10b levels.

## Figures and Tables

**Table 1:** Demographic data and baseline characteristics of study patients.

Calcitriol Use in Past Three Months (Total *n* = 225)	No (*n* = 81)	Yes (*n* = 144)	*p* Value
Age	69.86 ± 12.12	68.47 ± 12.21	0.37
Male	22 (33.8%)	76 (47.5%)	0.35
PTH (pg/mL)	358.12 ± 417.99	530.64 ± 481.73	<0.01 **
ALP (IU/L)	109.52 ± 82.72	121.51 ± 65.49	0.03 *
Calcium (mg/dL)	8.82 ± 0.66	8.68 ± 0.78	0.19
Phosphate (mg/dL)	4.51 ± 1.14	4.84 ± 1.33	0.07
Albumin (g/dL)	3.53 ± 0.39	3.40 ± 0.40	0.01 **
Hemoglobin (g/dL)	10.48 ± 1.30	10.28 ± 1.59	0.33
FGF-23 (pg/mL)	123.43 ± 166.52	452.85 ± 776.40	<0.01 **

Values are mean ± standard deviation if normally distributed. Value are medians ± interquartile ranges if not normally distributed. Abbreviations: PTH, Parathyroid hormone; ALP, Alkaline phosphatase; FGF-23, Fibroblast growth factor 23. * *p* < 0.05, ** *p* < 0.01.

**Table 2:** Bone turnover markers according to the use of calcitriol in hemodialysis patients.

Calcitriol Use in Past Three Months (Total *n* = 225)	No (*n* = 81)	Yes (*n* = 144)	*p* Value
Wnt 10b (ng/mL)	2.72 ± 1.26	4.13 ± 1.08	<0.01 **
Wnt 16 (pg/mL)	44.62 ± 53.84	36.76 ± 38.83	0.45
β-catenin (ug/mL)	7.86 ± 5.32	8.86 ± 6.26	0.20
Sclerostin (pg/mL)	89.87 ± 59.53	72.10 ± 43.12	0.06
DKK-1 (pg/mL)	232.72 ± 449.46	142.11 ± 277.62	0.03 *
Trap5b (U/L)	10.13 ± 4.04	5.57 ± 3.41	<0.01 **
Free RANKL (pmol/L)	0.08 ± 0.07	0.08 ± 0.07	0.997
OPG (pmol/L)	16.88 ± 6.90	16.71 ± 8.56	0.58
P1NP (pg/mL)	43.04 ± 44.27	158.87 ± 151.95	<0.01 **

Values are mean ± standard deviation if normally distributed. Value are medians ± interquartile ranges if not normally distributed. Abbreviations: DKK-1: Dickkopf-related protein 1; RNAKL: Receptor activator of nuclear factor kappa-B ligand; Trap5b: Tartrate-resistant acid phosphatase 5b; OPG: Osteoprotegerin; P1NP: Procollagen 1 N-terminal Propeptide. * *p* < 0.05, ** *p* < 0.01.

**Table 3:** The correlation coefficient in hemodialysis patients receiving calcitriol in the past three months.

Bone Turnover Markers	Wnt 10b	Wnt 16	β-catenin	Sclerostin	DKK-1	Trap5b	Free RANKL	OPG	P1NP	PTH	FGF23	25-(OH)D
**Wnt 10b**	1	0.351 **	0.083	−0.188 *	0.050	−0.450 **	0.038	−0.068	0.593 **	0.255 **	0.316 **	−0.019
**Wnt 16**	0.351 **	1	−0.099	−0.181 *	0.055	−0.351 **	0.069	−0.058	0.402 **	0.047	0.216 **	−0.075
**β-catenin**	0.083	−0.099	1	−0.140	0.033	−0.167	−0.032	0.019	−0.231 **	0.091	−0.190 *	0.084
**Sclerostin**	−0.188 *	−0.181 *	−0.140	1	−0.032	0.160	−0.041	−0.089	−0.150	−0.136	−0.188 *	0.136
**DKK-1**	0.050	0.055	0.033	−0.032	1	−0.152	−0.022	0.144	0.107	−0.206 *	0.219 *	−0.196 *
**Trap5b**	−0.450 **	−0.351 **	−0.167	0.160	−0.152	1	0.078	−0.027	−0.303 **	0.343 **	−0.168	0.022
**Free RANKL**	0.038	0.069	−0.032	−0.041	−0.022	0.078	1	−0.219 *	0.029	−0.043	0.004	0.033
**OPG**	−0.068	−0.058	0.019	−0.089	0.144	−0.027	−0.219 *	1	−0.050	−0.243 **	−0.084	−0.057
**P1NP**	0.593 **	0.402 **	−0.231 **	−0.150	0.107	−0.303 **	0.029	−0.050	1	0.209 *	0.521 **	−0.128
**PTH**	0.255 **	0.047	0.091	−0.136	−0.206 *	0.343 **	−0.043	−0.243 **	0.209 *	1	0.062	0.102
**FGF23**	0.316 **	0.216 **	−0.190 *	−0.188 *	0.219 *	−0.168	0.004	−0.084	0.521 **	0.062	1	−0.148
**25-(OH)D**	−0.019	−0.075	0.084	0.136	−0.196 *	0.022	0.033	−0.057	−0.128	0.102	−0.148	1

Abbreviations: DKK-1: Dickkopf–related protein 1; RNAKL: Receptor activator of nuclear factor kappa-B ligand; Trap5b: Tartrate-resistant acid phosphatase 5b; OPG: Osteoprotegerin; P1NP: Procollagen I N-terminal Propeptide; PTH: Parathyroid hormone; FGF-23: Fibroblast growth factor 23; 25-(OH)D: 25-hydroxyvitamin D. * *p* < 0.05, ** *p* < 0.01.
